# Preparation of NIR-Responsive Gold Nanocages as Efficient Carrier for Controlling Release of EGCG in Anticancer Application

**DOI:** 10.3389/fchem.2022.926002

**Published:** 2022-06-02

**Authors:** Weiran Gao, Xiangyi Fan, Yunlong Bi, Zipeng Zhou, Yajiang Yuan

**Affiliations:** ^1^ Department of Oncology, First Affiliated Hospital of Jinzhou Medical University, Jinzhou, China; ^2^ Department of Otolaryngology-Head and Neck Surgery, First Affiliated Hospital of Jinzhou Medical University, Jinzhou, China; ^3^ Department of Orthopedics, First Affiliated Hospital of Jinzhou Medical University, Jinzhou, China

**Keywords:** gold nanocages, epigallocatechin gallate, photothermal therapy, NIR responsive, hepatocellular carcinoma

## Abstract

Hepatocellular carcinoma (HCC) is a type of cancer that has a restricted therapy option. Epigallocatechin gallate (EGCG) is one of the main biologically active ingredients in tea. A large number of studies have shown that EGCG has preventive and therapeutic effects on various tumors. In addition, the development of near-infrared (NIR)-responsive nano-platforms has been attracting cancer treatment. In this work, we designed and synthesized a strategy of gold nanocages (AuNCs) as an efficient carrier for controlling release of EGCG for anti-tumor to achieve the synergistic functions of NIR-response and inhibited tumor cell proliferation. The diameter of AuNCs is about 50 nm and has a hollow porous (8 nm) structure. Thermal imaging-graphic studies proved that the AuNCs-EGCG obtained have photothermal response to laser irradiation under near-infrared light and still maintain light stability after multiple cycles of laser irradiation. The resulted AuNCs-EGCG reduced the proliferation rate of HepG2 cells to 50% at 48 h. Western blot analysis showed that NIR-responsive AuNCs-EGCG can promote the expression of HepG2 cell apoptosis-related proteins HSP70, Cytochrome C, Caspase-9, Caspase-3, and Bax, while the expression of Bcl-2 is inhibited. Cell confocal microscopy analysis proved that AuNCs-EGCG irradiated by NIR significantly upregulates Caspase-3 by nearly 2-fold and downregulates Bcl-2 by nearly 0.33-fold, which is beneficial to promote HepG2 cell apoptosis. This study provides useful information for the NIR-responsive AuNCs-EGCG as a new type of nanomedicine for HCC.

## Introduction

Hepatocellular carcinoma (HCC) is a malignant tumor of the liver. It is a malignant tumor with high incidence and great harm in the world (Marrero et al., 2018). According to the different stages of HCC, individualized comprehensive treatment is the key to improving the efficacy. Surgery, hepatic artery ligation, hepatic artery chemoembolization, radiofrequency, cryo, laser, microwave, chemotherapy, and radiotherapy are some of the treatment options (Cammarota et al., 2021; Carballo-Folgoso et al., 2021; Hu et al., 2021a; Monge et al., 2021; Su et al., 2021). HCC is also treated with biological therapy and traditional Chinese medicine (Chang et al., 2021; Luo et al., 2021; Oura et al., 2021; Sorop et al., 2021). These conventional therapies have limitations such as side effects, low bioavailability, and poor efficacy. Therefore, it is of great significance to find a new treatment method to reduce side effects and even increase anti-tumor activity.

With the development of medicine and the progress of chemotherapy, photothermal therapy (PTT) has gradually attracted people’s attention. Its low side effects and high efficiency make it an emerging cancer treatment method (Zheng et al., 2020; Chang et al., 2021; Cui et al., 2021; Shen et al., 2021). Photothermal responses are exploited as nanoplatforms to promote bacterial infection wound healing and treat arthritis (Ding et al., 2021; Xu et al., 2021; Xu et al., 2022). Gold has good physical tolerance and ductility, and the particularity of releasing heat after absorbing light has become a hot spot for oncologists (Goddard et al., 2020; Mirzadeh et al., 2021). Gold nanoparticles have been shown to be effective in the treatment of cancers in studies. When a light source is used to illuminate the nanoparticles, heat is released, which kills cancer cells nearby (Cheng et al., 2021; He et al., 2021; Medici et al., 2021). Compared with solid gold nanoparticles whose plasmon resonance is realized only on the outer surface, gold nanocages (AuNCs) are hollow inside, which have higher anticancer drug loading capacity, higher photothermal conversion efficiency, and near-infrared region (Hubert et al., 2021). The tunable localized surface plasmon resonance (LSPR) band and excellent biocompatibility make it an ideal carrier for cancer control (Sun et al., 2020). Because AuNCs have plasmon resonance absorption on both the outer and inner surfaces, they can be used as a better light-to-heat conversion agent to induce tumor apoptosis by overheating (Fang et al., 2020; Hu et al., 2021b). In addition, the porous structure on the surface of AuNCs greatly increases the “hot spots” as a surface-enhanced Raman scattering and substrate (Jindal et al., 2020). Furthermore, the electromagnetic field enhancement effect caused by the superimposed plasmon resonance on the inner and outer surfaces of AuNCs makes it a wide range of the promising surface-enhanced Raman scattering substrate. Which aims to achieve single-molecule detection in the liquid phase *via* surface-enhanced Raman scattering (Farahavar et al., 2021). In addition, compared to Au nanorods and nanoshells, AuNCs have large free volume to be an ideal drug carrier.

EGCG (epigallocatechin gallate) as a natural active compound has shown anti-tumor activity *in vitro* or *in vivo* experiments (Bhat and Pezzuto, 2002; Singh et al., 2011; Ren et al., 2020). EGCG has antioxidant, antibacterial and immune enhancing, anticancer, etc., which has attracted much attention (Chen et al., 2013; Xing et al., 2019; Li et al., 2021). EGCG and other chemotherapeutics such as gefitinib (Abe et al., 2018) and bleomycin (Alshatwi et al., 2016) can reduce drug dosage and drug resistance, and show good synergy. Researchers began to try to use these compounds as anti-cancer adjuvants to enhance the anti-tumor activity of clinical chemotherapeutics (Lecumberri et al., 2013; Zhang et al., 2019). However, the application of EGCG in cancer therapy faces obstacles due to its low stability, low bioavailability, poor absorption, and rapid elimination. Therefore, a carrier capable of efficiently loading and releasing EGCG is urgently needed to solve this problem.

**SCHEME 1:**
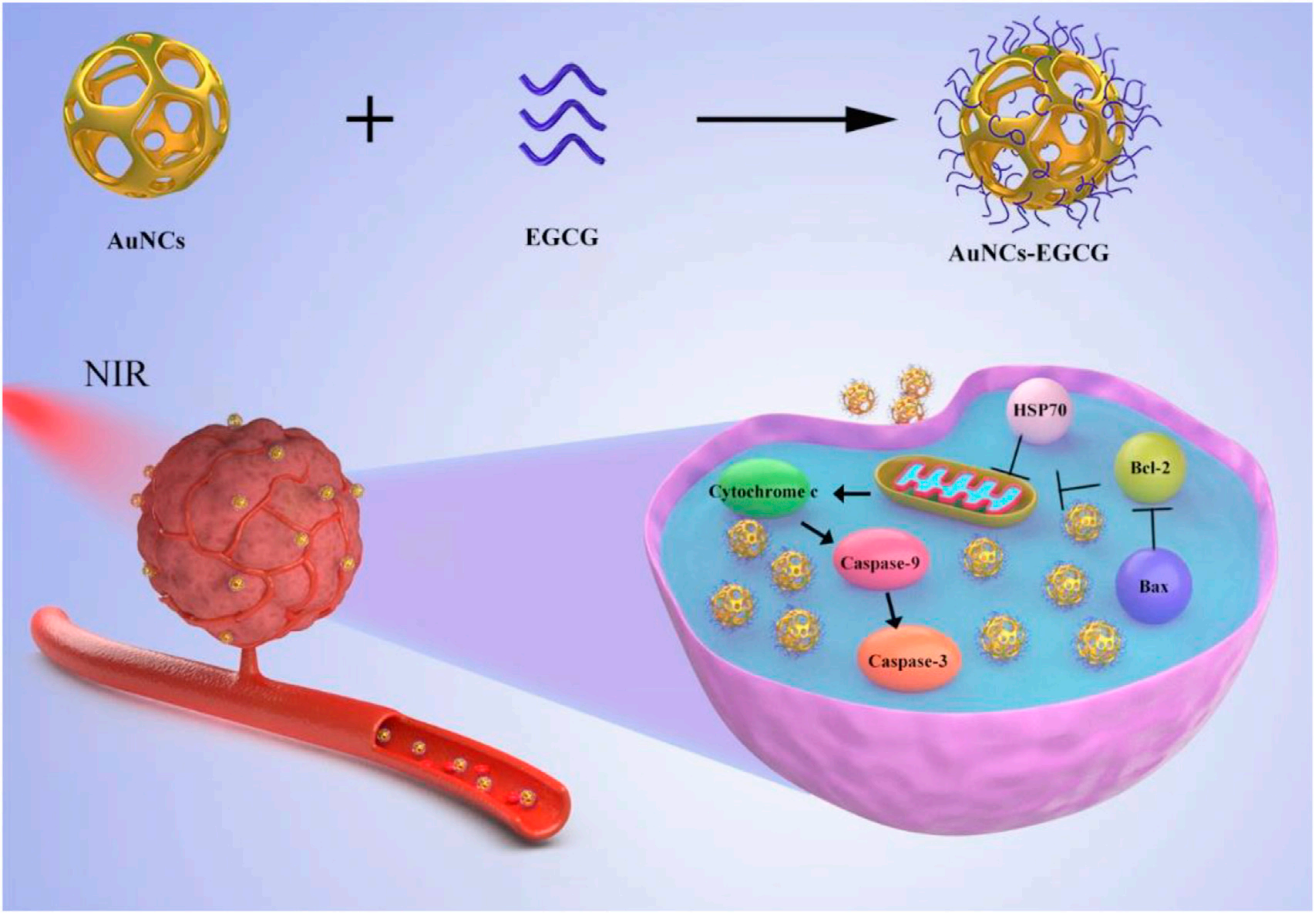
Schematic diagram of the synthesis of AuNCs-EGCG and its effect on cells under near-infrared radiation. AuNCs-EGCG can promote tumor cell apoptosis.

In this article, to achieve the synergistic functionalities of NIR-response and decreased tumor cell proliferation, we devised and synthesized an approach of employing EGCG modified AuNCs as a therapeutic nanoplatform for anti-tumor (Scheme 1). We found that AuNCs-EGCG can effectively inhibit the proliferation efficiency of HepG2 cells through photothermal therapy. And significantly increase the expression level of apoptosis-related proteins and induce cell apoptosis. This study provides useful information for NIR-responsive AuNCs-EGCG as a new type of nanomedicine for liver cancer.

## Materials and Methods

### Materials

Dulbecco’s modified Eagle’s medium (DMEM), fetal bovine serum (FBS), and 3-(4,5-Dimethylthiazol-2-yl)-2,5-diphenyl tetrazolium bromide (MTT) were purchased from Gibco (United States). Dimethyl sulfoxide (DMSO) was purchased from Sigma-Aldrich (United States). RIPA lysis buffer and phenylmethanesulfonyl fluoride (PMSF) were obtained from Solarbio (China). The primary antibodies to cytochrome C, Bax, Bcl-2, caspase-3, caspase-9, HSP70, β-Tubulin, and β-actin were purchased from Cell Signaling Technology (United States). The secondary antibodies to HRP AffinPure goat anti-rabbit IgG and HRP AffinPure goat anti-mouse IgG were purchased from Proteintech (United States). The Alexa Fluor®488 goat anti-mouse/rabbit IgG and Alexa Fluor®568 goat anti-mouse/rabbit IgG were purchased from Invitrogen (United States). Triton X-100 and 4,6-dimethyl-2-phenylindole (DAPI) were purchased from Abcam (United Kingdom). Human hepatocellular carcinomas (HepG2) were obtained from American type culture collection (ATCC). The 2,2’-bis(anthracene-9,10-diylbis (methylene))-dimalonic acid (ABDA) was obtained from Shanghai Civi Chemical Technology Co.

### Preparation of AuNCs and AuNCs-Epigallocatechin Gallate

The three-necked flask was first filled with 10 ml of PVP (4 mg/ml), followed by 4 ml of silver nanoparticles. The solution was then heated in an oil bath (100°C, 500 r/min). After 15 min, HAuCl4 (0.0543 mg/ml) were added until the color was stable. To eliminate contaminants, the samples were washed with saturated NaCl solution and deionized water after the temperature was decreased to 25°C. AuNCs were obtained in this manner. The next step was to combine 1 mg EGCG with 1 ml AuNCs and leave the combination overnight to create a clear solution.

### Characterization

A transmission electron microscope (TEM, JEM-1200EX, Tokyo, Japan) was used to examine the morphology of AuNCs. Dynamic laser scattering (DLS, Malvern, NanoZS90, Worcestershire, United Kingdom) was used to determine the size of AuNCs. Ultraviolet-visible spectrophotometer (PerkinElmer Lambda 605S, Waltham, MA, United States) and fluorophotometer (F97PRO, Shanghai, China) obtained ultraviolet and fluorescence data respectively.

### Drug Release From AuNCs-Epigallocatechin Gallate

AuNCs-EGCG was added in PBS (pH 7.4) for *in vitro* release assay. AuNCs-EGCG was dissolved in buffer solution and the solution was placed in a dialysis bag (Spectra/Por Float-A-Lyzer G2, United States). Fill the sealed dialysis bag with 50 ml of release medium (PBS, pH = 7.4) and incubate at 37°C with gentle shaking (100 r/min). Different groups will be irradiated or not irradiated with NIR light, and 4 ml of the solution will be removed from the centrifuge tube at intervals for UV-vis spectroscopy analysis while adding the same volume of buffer to the system.

### Photocatalytic Performance of AuNCs-Epigallocatechin Gallate of AuNCs-Epigallocatechin Gallate

ABDA probes were used to detect singlet oxygen production and changes over time. Fluctuations of the ABDA probe were recorded to reflect ROS production. We added AuNCs-EGCG to a 5 mM ABDA solution and then irradiated it under a near-infrared laser. This test is performed every 5 min. Furthermore, the resulting single linear oxygen state was detected by electron spin resonance spectroscopy.

### Cell Culture and Treatment

In a humidified incubator at 37°C and 5% CO2, HepG2 cells were grown in DMEM medium supplemented with 1% penicillin-streptomycin and 10% FBS. The cells were split into two groups for treatment: the group in charge (the cells were not treated) and the photothermal response AuNCs-EGCG group (with 50 μg/ml AuNCs-EGCG and laser irradiation for 2 h).

### Cell Viability Assay

The MTT assay was used to detect cell viability. Inoculate the cells (5,000/well) in a 96-well plate. After adding 50 μg/ml AuNCs-EGCG and laser irradiation for 2 h, the cells were cultured for 12, 24, 48, and 72 h. Then, over the next 4 h, 20 μl of MTT solution (5 mg/ml in PBS) was put to each well in the incubator. After that, 150 ml DMSO was added to each well and incubated for 10 min at 37°C. Finally, a microplate reader was used to measure the absorbance at 490 nm.

HepG2 cells were incubated with AuNCs (50 μg/ml) or AuNCs-EGCG (50 μg/ml) for 24 h. And treated with NIR laser for 3 min. Cells were stained with CalceinAM/PI mixed fluorescent dye for 10 min to label live and dead cells. Finally, we used a fluorescent microscope to image live/dead cells.

### Western Blot Analysis

HepG2 cells were collected and lysed for 20 min on ice in RIPA lysis solution containing PMSF, before being dissociated using an ultrasonic homogenizer. To separate the protein supernatant, the treated cells were centrifuged at 12,000 rpm for 25 min at 4°C. The proteins were electrophoretically separated and transferred to a PVDF membrane, which was subsequently blocked for 2 h at room temperature with 5% skimmed milk powder. The cells were then treated overnight at 4°C with the primary antibodies listed below: cytochrome C (1:1,000), Bax (1:1,000), Bcl-2 (1:1,000), Caspase-3 (1:1,000), Caspase-9 (1:1,000), HSP70 (1:1,000), and β-actin (1:1,000). After washing the membrane for 3 min × 5 min with TBST, secondary antibodies were used to incubate the membrane: HRP AffinPure goat anti-rabbit IgG (1:10,000) and HRP AffinPure goat anti-mouse IgG. (1:10,000). Beyo ECL Plus was used to visualize immune response bands, which were then quantified using grayscale analysis and ImageJ2x software.

### Confocal Fluorescence Imaging

In a confocal Petri plate, HepG2 cells were grown, washed three times with PBS, then infiltrated with 0.1 percent Triton X-100 for 30 min. The cells were blocked with normal goat serum for 2 h before being washed three times with 0.1% PBS. Anti-Tubulin (1:1,000), anti-caspase-3 (1:200), and anti-Bcl-2 (1:200) primary antibodies were added and incubated at 4°C overnight. The cells were washed three times with 0.1% PBS the next day. Then, Alexa Fluor®568 goat anti-mouse/rabbit IgG (1:500) and Alexa Fluor®488 goat anti-mouse/rabbit IgG (1:500) were added and incubated for 2 h at room temperature. The cells were rinsed with 0.1% PBS (3 min × 5 min) and incubated with DAPI (1:1,000) for 30 min. Finally, an observation was performed using a high-resolution confocal microscope.

### Statistical Analysis

SPSS 19.0 was used to do statistical analysis on all of the collected data, which was expressed as mean standard deviation (SD). The two groups were compared using one-way analysis of variance (ANOVA). Statistical significance was defined as a *p* value of less than 0.05.

## Results and Discussion

### Characterization of the Materials

**FIGURE 1:**
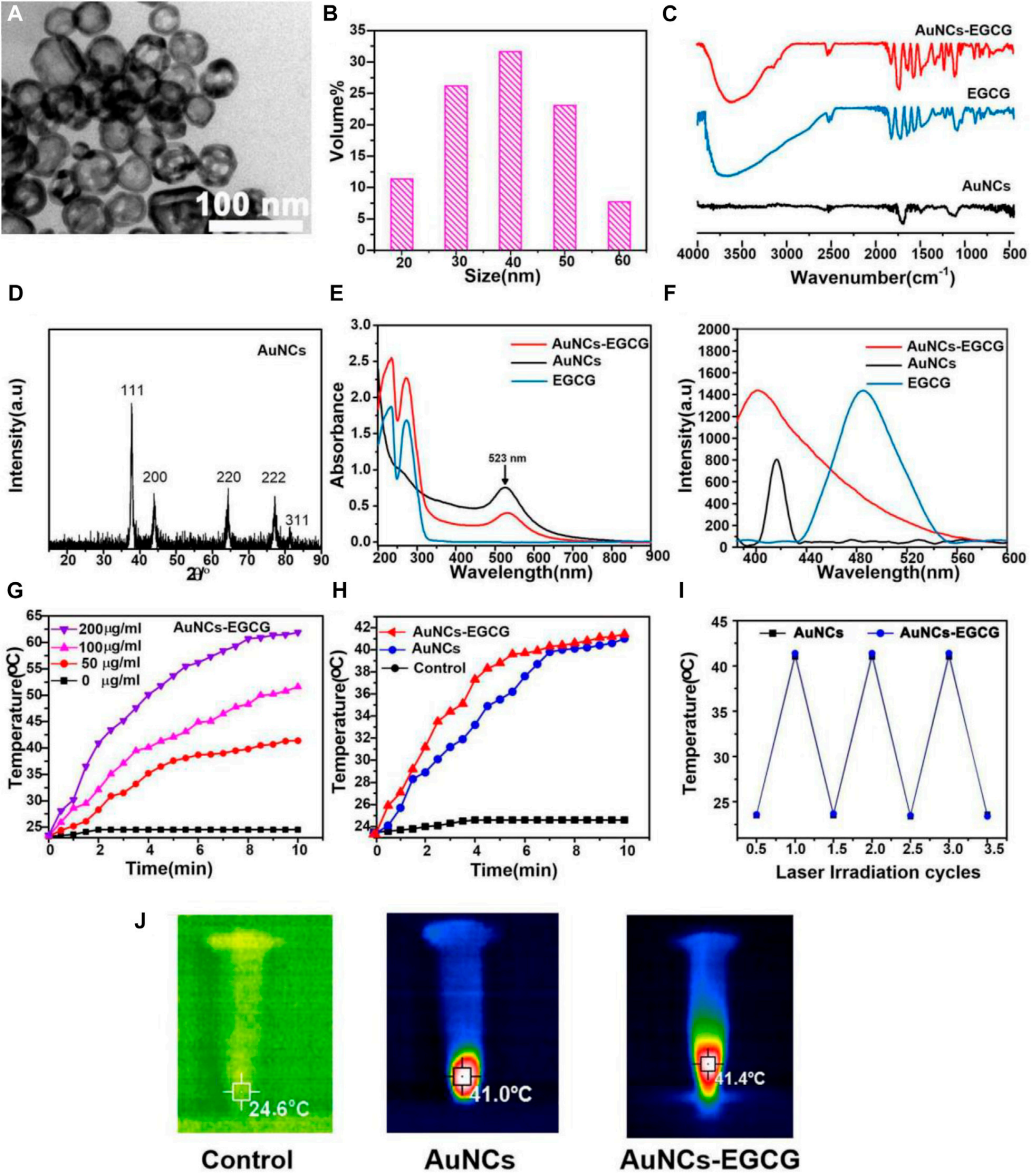
Characterization of AuNCs and EGCG-AuNCs. **(A)** TEM images and **(B)** DLS size distribution of AuNCs. **(C)** FTIR spectra, **(D)** XRD, **(E)** UV-vis and **(F)** fluorescence emission (λex = 400 nm) of samples. **(G)** Temperature rise curves of AuNCs-EGCG at different concentrations within 10 min. **(H)** Temperature rise curves of three groups of samples within 10 min. **(I)** The thermal curves of AuNCs and EGCG-AuNCs after repeated laser irradiation (*n* = 3). **(J)** Infrared thermography images of samples under 808 nm laser irradiation for 5 min.

The morphology and size distribution of the prepared AuNCs were characterized by TEM and DLS, respectively. As shown in Figure 1A, we observed the morphology of AuNCs by TEM. They had a well-shaped porous hollow structure (8 nm) and uniform size (50 nm). In Figure 1B, the diameter of AuNCs was about 50 nm through DLS detection. These AuNCs had a polydispersity index (PDI) of 0.1, indicating that they were uniform. The absorption peak at 1,725 cm^−1^ and the broad absorption peak at 1,200–1,700 cm^−1^ were characteristic absorption peaks of –C=O and benzene of EGCG, while 1,100 cm^−1^ and 1,250 cm^−1^ were P=O stretching vibration peaks, according to FTIR spectra. It was established that the AuNCs-EGCG that had been manufactured already contained EGCG (Figure 1C). The diffraction peaks at 45°, 64°, and 76° in Figure 1D correlated closely to the diffraction peaks of AuNCs’ crystal planes (200), (220), and (222), indicating that AuNCs retains its full structure when linked to EGCG. The synthesis of AuNCs-EGCG and AuNCs was confirmed by UV-vis (Figures 1E,F). At 523 nm, there is a distinctive peak with a broad absorption range, indicating that the AuNCs have a homogeneous particle size distribution and regular morphology, which is consistent with the characterisation results. The temperature changes of AuNCs-EGCG and AuNCs solutions at varied concentrations (0, 50, 100, and 200 g/ml) during NIR laser irradiation were then monitored to determine the photothermal effect of AuNCs, and the highest temperature infrared image was obtained. As shown in Figure 1G, when the concentration of AuNCs-EGCG reaches 200 μg/ml, the temperature can be as high as 63.2°C after 8 min of near-infrared light irradiation, while the temperature of AuNCs-EGCG rises faster under the same irradiation conditions (Figure 1H). These findings suggested that AuNCs-EGCG could be a potent photothermal agent. As shown in Figure 1H, AuNCs (50 μg/ml) and AuNCs-EGCG (50 μg/ml) were used to compare the difference in temperature rise between the two group under the same conditions. It can be shown that AuNCs-EGCG has a faster temperature rise, indicating that AuNCs-EGCG has strong photothermal conversion capabilities. The heat production efficiency of AuNCs and AuNCs-EGCG was tested after three heating and cooling process cycles in order to assess the photothermal conversion stability of AuNCs-EGCG. The temperature rise of AuNCs and AuNCs-EGCG did not differ significantly after three cycles, as shown in Figure 1I, indicating AuNCs-outstanding EGCG’s photothermal stability. In addition, as detected in Figure 1J, we intuitively observed that their maximum temperature could rise to 41.4°C, which was enough to cause irreversible cell damage. In conclusion, AuNCs-EGCG has a stronger photothermal impact, making it useful for mediating PTT without using ultra-high-density lasers.

**FIGURE 2:**
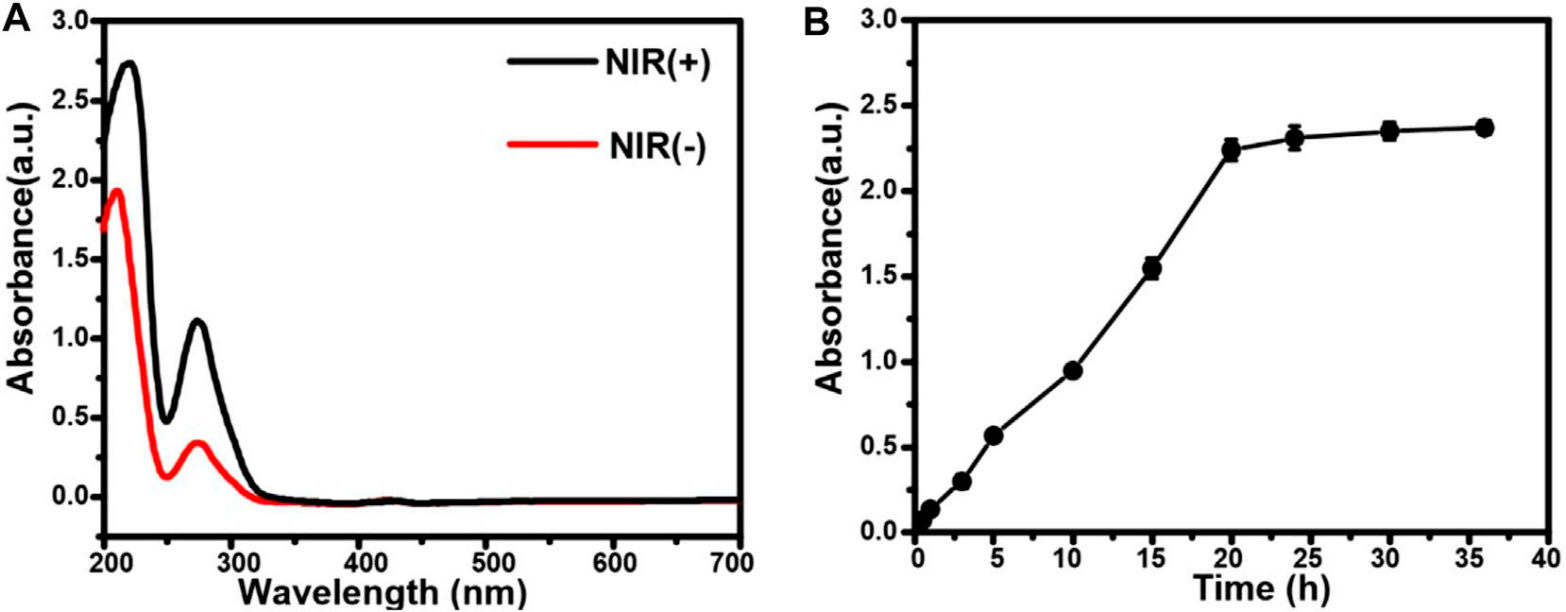
Drug release from AuNCs-EGCG. **(A)** The release of EGCG from AuNCs-EGCG with or without laser irradiation. **(B)** The drug release curve of AuNCs-EGCG.

The release of EGCG from AuNCs-EGCG under near-infrared laser irradiation was evaluated by UV-vis spectrophotometry and dialysis bag method. As shown in Figure 2A, AuNCs-EGCG released more EGCG under 808 nm laser irradiation, suggesting that NIR laser irradiation could modulate the drug release. The results of *in vitro* drug release test showed that the release amount of EGCG in EGCG-AuNCs increased with time, but the release rate tended to be gentle after 20 h, and the release amount did not change significantly (Figure 2B).

**FIGURE 3:**
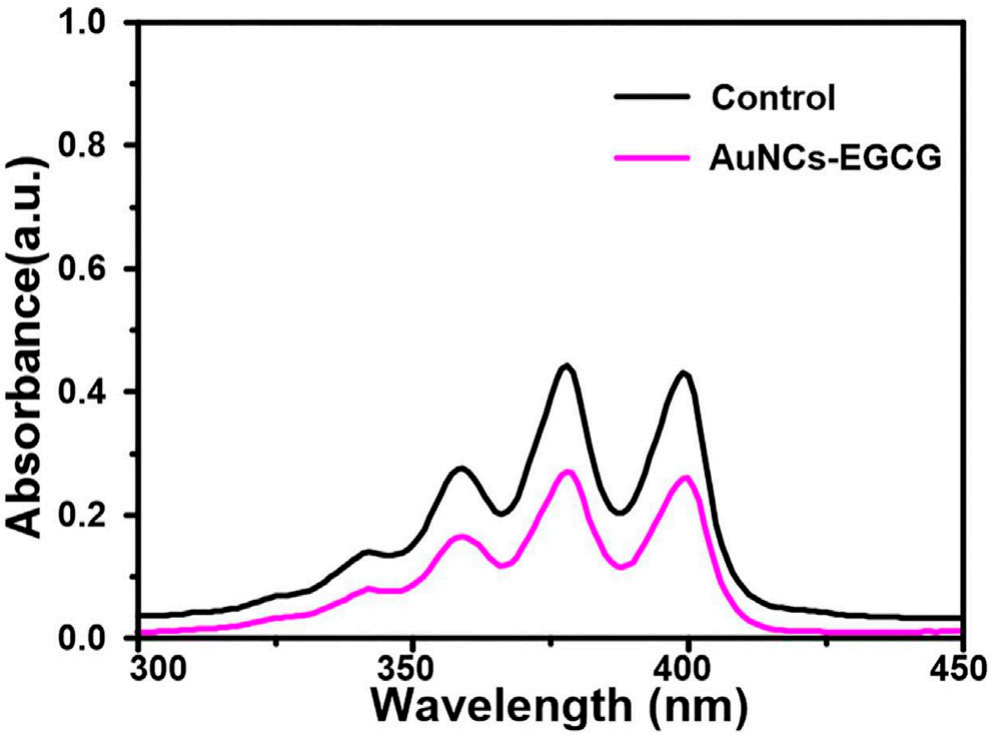
UV spectra of ABDA solution in control group and AuNCs-EGCG group.

The ability of AuNCs-EGCG to generate singlet oxygen under near-infrared laser irradiation was evaluated using ABDA as an indicator. As shown in Figure 3, the absorbance at 380 nm of the ABDA solution in the presence of AuNCs-EGCG was significantly decreased compared with the control group, indicating that AuNCs-EGCG was able to generate singlet oxygen under 808 nm laser irradiation.

### Effect of Near-Infrared-Responsive AuNCs-Epigallocatechin Gallate on Cell Proliferation *In Vitro*

**FIGURE 4:**
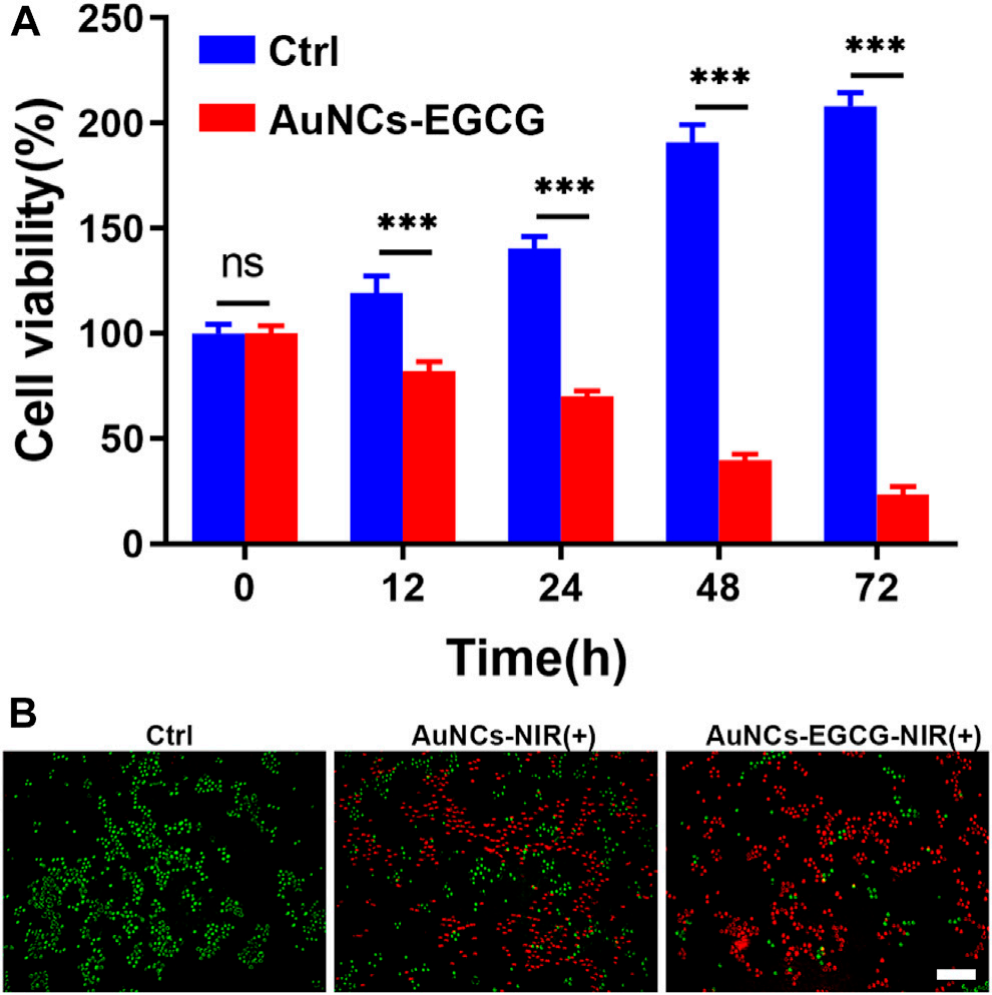
NIR-responsive AuNCs-EGCG inhibited proliferation of HepG2 cells. **(A)** Effects of NIR-responsive AuNCs-EGCG at different times on the proliferation of HepG2 cells observed by MTT method. The mean and standard deviation (SD) of the data (*n* = 3) are shown. Statistical analysis: ^∗∗∗^ *p* < 0.001. **(B)** Fluorescent images of Live/Dead-stained HepG2 cells after different treatments. Green: live cell staining, Red: dead cell staining. Scale bars are 25 μm.

Tumors refer to cells that have undergone genetic changes under the action of tumorigenic factors and lose their normal regulation of their growth, leading to abnormal proliferation (Shen et al., 2020). Malignant tumors grow rapidly and can metastasize to other parts of the body. They can also produce harmful substances, destroy normal organ structures, make the body dysfunction, and threaten lives (Liu et al., 2021). Cancer cells have three significant basic characteristics: immortality, migration, and loss of contact inhibition (Nia et al., 2020). In addition, cancer cells have many physiological, biochemical, and morphological characteristics that are different from normal cells. Cancer cells vary in size and shape, and are usually larger than their source cells and grow faster. Effectively inhibiting the proliferation of cancer cells will help the treatment of tumors (Gnocchi et al., 2021). To prove the effect of Near-Infrared (NIR)-responsive AuNCs-EGCG on HepG2 cells proliferation. We used the MTT method to evaluate the proliferation efficiency of the cells after 12, 24, 48, and 72 h in culture (Figure 4A). We found that after 2 h of treatment with laser-irradiated AuNCs-EGCG, the cell proliferation rate gradually decreased with time. And at 48 h, the cell proliferation rate was reduced to less than 50%. This result indicated that NIR-responsive AuNCs-EGCG has obvious cytotoxicity and significantly inhibited the proliferation of HepG2 cells.

We assessed and compared the photothermal effects of AuNCs and AuNCs-EGCG by live/dead cell staining. As shown in Figure 4B, the control group only showed green color, meaning that there were almost no dead cells. As expected, different degrees of red fluorescence were observed in HepG2 cells in the NIR group. Compared with the sporadic weak red fluorescence of the AuNCs group, the AuNCs-EGCG group showed obvious antitumor ability, and the strongest uniform red fluorescence meant that almost no cells survived. These results demonstrate that surface AuNCs-EGCG has a better therapeutic effect.

### Effect of Near-Infrared-Responsive AuNCs-Epigallocatechin Gallate on Cell Apoptosis *In Vitro*

Apoptosis is also called “pyknotic necrosis”, “programmed cell death” or “cell suicide” (Fumarola and Guidotti, 2004; Jin and El-Deiry, 2005). It is a series of changes mediated by genes, and cells rely on it to actively cause its own destruction (Debatin, 2004; Ghobrial et al., 2005). Normally, it eliminates the apoptotic process of aging cells or lymphocytes that are not involved in the immune response (Eichhorst, 2005). If pathological interference occurs, it can play a role in tumor formation (Ghobrial et al., 2005). Apoptosis is characterized by the tendency of cells to die in an almost normal plasma membrane due to degrading enzymes, mainly hydrolases (proteases and nucleases) (Basu, 2022). It can be physiological, or induced by chemotherapy drugs and radiation. It is a self-destruct mechanism exists in cells. During this process, the body can eliminate senescent and abnormal cells, and plays an important role in maintaining many cell functions. Normal cells start the process of apoptosis when their chromosomes change, but in cancer cells, the signal pathways related to apoptosis are blocked, which means that cancer cells are immortal. Cancer cells with mutations turn off this regulatory function of the mitochondria inside the cell, so they escape this regulatory mechanism and do not self-destruct (Gastman, 2001; Shirjang et al., 2019; Wong et al., 2019).

**FIGURE 5:**
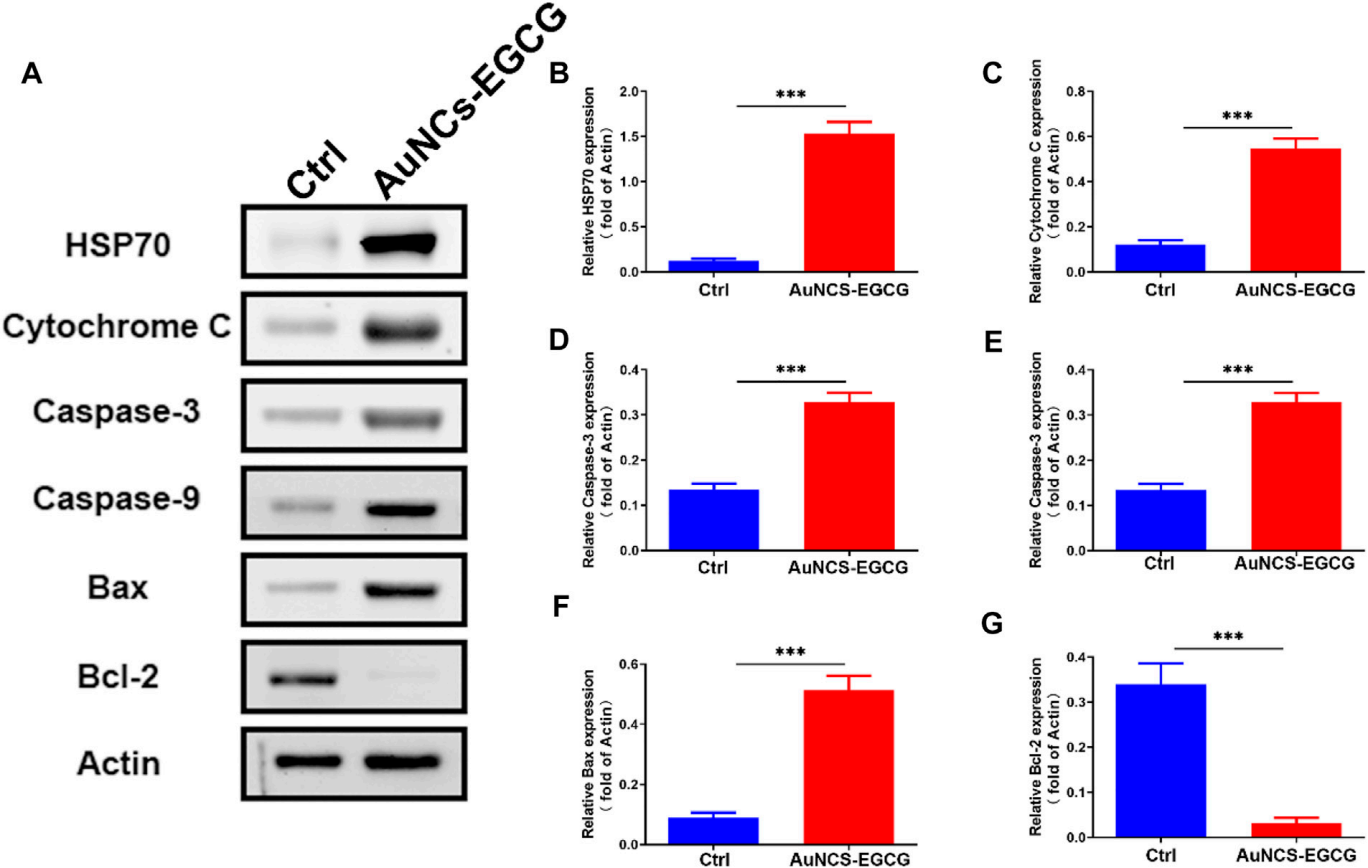
NIR-responsive AuNCs-EGCG promoted HepG2 apoptosis. **(A–G)** The effect of NIR-responsive AuNCs-EGCG on the expression of HepG2 apoptosis-related proteins was detected by western blot and semi-quantitative analysis. The mean and standard deviation (SD) of the data (*n* = 3) are shown. Statistical analysis: ^∗∗∗^ *p* < 0.001.

**FIGURE 6:**
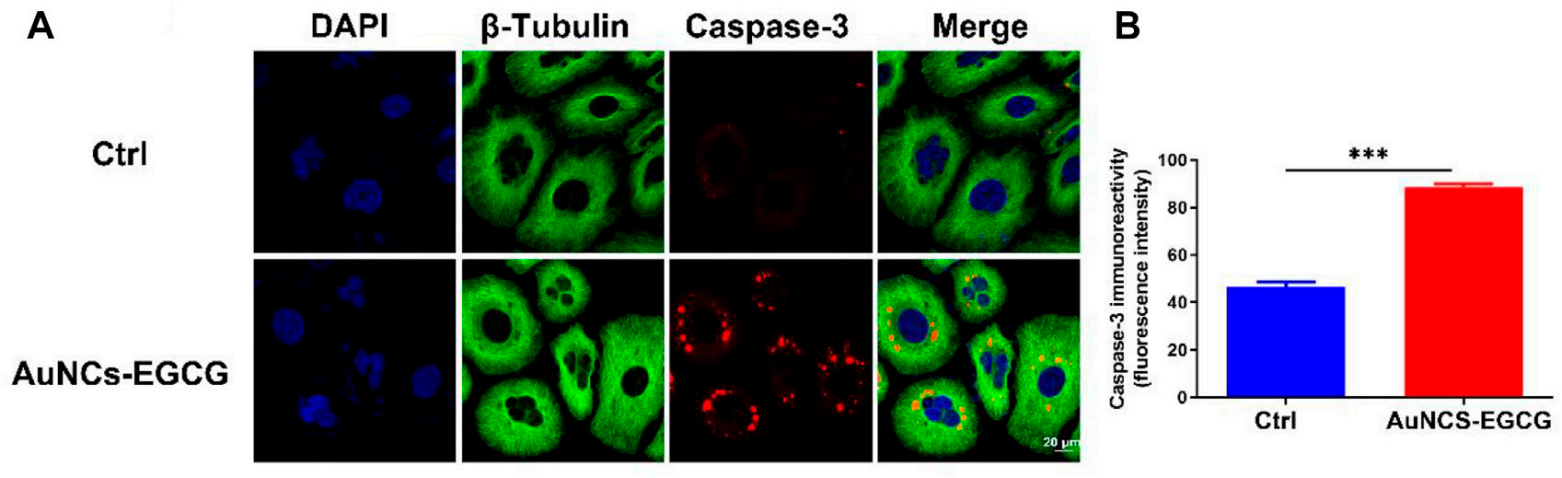
NIR-responsive AuNCs-EGCG promoted the expression level of Caspase-3 protein in HepG2 cells **(A,B)**. Confocal microscopy immunofluorescence analysis of Caspase-3 protein expression and its semi-quantification analysis. Scale bars are 25 μm. The mean and standard deviation (SD) of the data (*n* = 3) are shown. Statistical analysis: ^∗∗∗^ *p* < 0.001.

**FIGURE 7:**
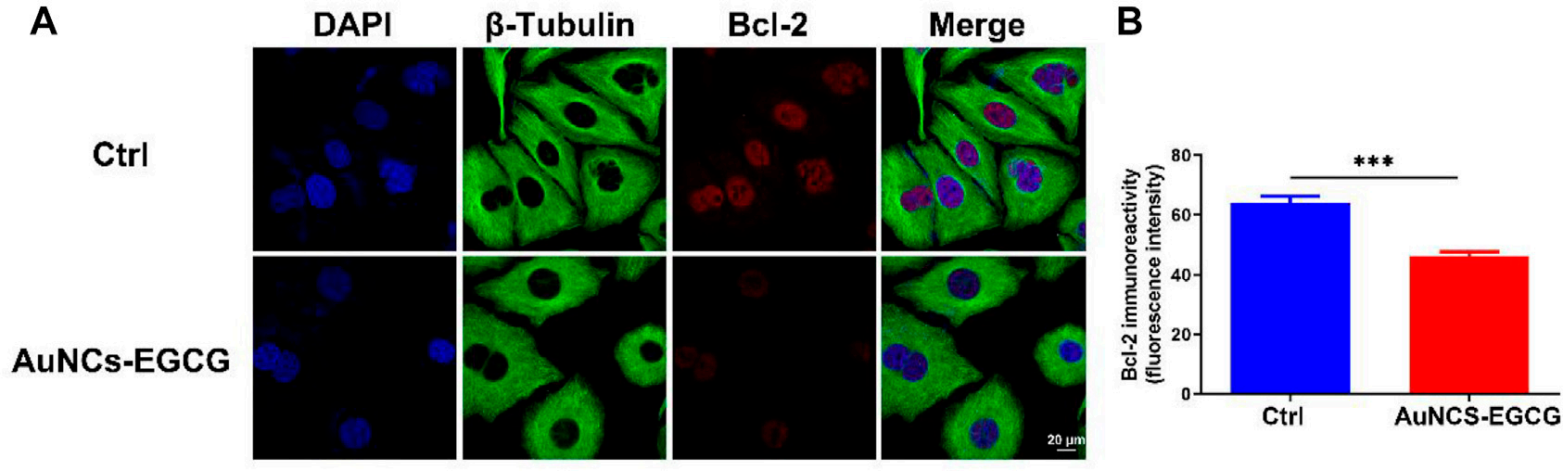
NIR-responsive AuNCs-EGCG inhibited the expression level of Bcl-2 protein in HepG2 cells **(A,B)**. Confocal microscopy immunofluorescence analysis of Bcl-2 protein expression and its semi-quantification analysis. Scale bars are 25 μm. The mean and standard deviation (SD) of the data (*n* = 3) are shown. Statistical analysis: ^∗∗∗^ *p* < 0.001.

Studies have shown that EGCG may interfere with the occurrence and development of cancer by inhibiting cell proliferation, inducing cell apoptosis, interfering with cell metabolism, inhibiting oncogene expression, and inhibiting tumor angiogenesis (Wang et al., 2018). In order to clarify the mechanism of AuNCs-EGCG promoting HepG2 cell apoptosis, after 72 h of cell culture, we used western blot analysis to check the expression of associated proteins. Figure 5 shows that following NIR response treatment, the expression level of heat shock protein (HSP70) was higher than in the control group, which indicated that HepG2 cells were experiencing high temperatures. This demonstrated that under near-infrared radiation, AuNCs-EGCG have a considerable thermal warming and killing impact. The AuNCs-EGCG group had considerably higher levels of Cytochrome C, Caspase-9, Caspase-3, and Bax expression than the control group. In contrast, the expression of the anti-apoptotic protein Bcl-2 was significantly downregulated. At the same time, we observed the expression levels of Caspase-3 and Bcl-2 using a cell confocal microscope. We found that AuNCs-EGCG increased the expression level of Caspase-3 to more than 50% (Figures 6A,B). At the same time, the expression level of Bcl-2 was dropped by 33.33% (Figures 7A,B), and these results were consistent with western blot. These findings showed that under NIR irradiation, AuNCs-EGCG can suppress HePG2 cell death via the mitochondrial pathway.

## Conclusion

In summary, we successfully designed and synthesized a EGCG modified AuNCs nanoplatform and studied the photothermal therapeutic effect of AuNCs-EGCG under NIR-responsive. This research may provide a new type of nanomedicine for HCC. Thermal imaging-graphic studies proved that the AuNCs-EGCG obtained have photothermal response to laser irradiation under near-infrared light and still maintain light stability after multiple cycles of laser irradiation. The resulted AuNCs-EGCG reduced the proliferation rate of HepG2 cells to 50% at 48 h. Western blot analysis showed that NIR-responsive AuNCs-EGCG can promote the expression of HepG2 cell apoptosis-related proteins HSP70, Cytochrome C, Caspase-9, Caspase-3, and Bax, while the expression of Bcl-2 is inhibited. Cell confocal microscopy analysis proved that AuNCs-EGCG irradiated by NIR significantly upregulates Caspase-3 by nearly 2-fold and downregulates Bcl-2 by nearly 0.33-fold, which is beneficial to promote HepG2 cell apoptosis. This study provides useful information for the NIR-responsive AuNCs-EGCG as a new type of nanomedicine for HCC.

## Data Availability

The raw data supporting the conclusion of this article will be made available by the authors, without undue reservation.
